# Benchmarking *Drosophila* receptor neurons for technical applications

**DOI:** 10.1186/1471-2202-13-S1-P155

**Published:** 2012-07-16

**Authors:** Thomas Nowotny, Stephen Trowell, Marien de Bruyne

**Affiliations:** 1 Engineering and Informatics, University of Sussex, Brighton, East Sussex, BN1 9QJ, UK; 2 Food Futures Flagship, CSIRO Eco Systems Sciences, Canberra, ACT 2601, Australia; 3 Biological Sciences, Monash University, Clayton, VIC 3800, Australia

## 

Animals detect volatiles in the environment with an animal-specific set of olfactory receptor molecules. The olfactory receptors (ORs) of the fruit fly *Drosophila melanogaster* form one of the experimentally best characterized sets of this kind. Recently, it has been suggested that, while the receptors have evolved to provide information about chemicals that are behaviorally relevant to the fly, they might also be used for technical applications [1]. Two quite disparate examples of envisioned applications are detecting the volatiles related to security threats [1], e.g. volatiles originating from explosives, and detecting and judging the volatiles related to wine making [2], e.g. to judge the quality of the resulting wine. It has been shown that *Drosophila* receptors show noticeable responses to the relevant chemicals in these applications [1,2] which underpins this idea. However, no systematic assessment has yet been performed of what type of problems *Drosophila* ORs are likely able to solve and which combination of ORs should be used to maximize the chances of success.

To address this problem we collected a large number of *in vivo* recordings from individual *Drosophila* olfactory receptor neurons in response to two sets of chemicals: A set of 36 chemicals related to wine making (“wine set”) [2] and a set of 35 chemicals related to security applications (“risk set”) [1]. We characterized the responses of olfactory receptor neurons, each expressing one of 20 considered OR types, by their mean firing rate and the standard deviation of the mean in repeated experiments. Due to the difficult experimental procedure not all chemicals can be measured for each neuron and the number of samples per neuron type varies significantly. To form one consistent set of responses, we, therefore, resampled 20 responses per OR type and chemical from a Gaussian distribution with the experimentally observed mean and standard deviation. We then used these response sets to classify the chemicals (odors) within the two sets all against all using a standard linear support vector machine classifier [3]. To address our original question of which combination of receptors promises the best odor recognition in the two example applications, we used a so-called wrapper approach: We formed all possible combinations of subsets of receptors and evaluated their performance in odor recognition using the linear SVM in 10-fold cross-validation.

We find that, in contrast to concurrent work with metal oxide sensors (Nowotny T, Berna A, Trowell S: *in prep.*), *Drosophila* receptors achieve the best recognition accuracy in both applications (81.5% for the wine set and 77.6% for the risk set) if the outputs of all 20 receptor types are used. However, a level of 90% of this performance (73.4% and 69.8% respectively) can already be achieved by an appropriately chosen set of only 10-11 receptors (The chance levels for the performance are 2.8% and 2.9% respectively). The sets of most relevant receptors for both applications have considerable overlap but are not identical. Interestingly, if only very few receptor types are utilized, *Drosophila* ORs distinguish the risk set chemicals significantly better than thos of the arguably behaviorally more relevant wine set. If all 20 receptor types are included, however, the situation is reversed and the wine set is classified better.

## Conclusions

Our computational analysis reveals that *Drosophila* receptors appear surprisingly capable to distinguish chemicals that they have not been evolved to process, making their use in technical applications a realistic possibility.
